# Manifestation of Generalized Anxiety Disorder and Its Association With Somatic Symptoms Among Occupational and Physical Therapists During the COVID-19 Pandemic

**DOI:** 10.3389/fpubh.2022.891276

**Published:** 2022-04-25

**Authors:** Musaed Z. Alnaser, Naser Alotaibi, Mohammed Sh. Nadar, Fahad Manee, Hesham N. Alrowayeh

**Affiliations:** ^1^Occupational Therapy Department, Faculty of Allied Health Sciences, Kuwait University, Kuwait City, Kuwait; ^2^Physical Therapy Department, Faculty of Allied Health Sciences, Kuwait University, Kuwait City, Kuwait

**Keywords:** psychological symptoms, physical symptoms, mental health, healthcare professionals, occupational therapists, physical therapists, anxiety, somatic

## Abstract

**Purpose:**

The purpose of the study was to examine the level of generalized anxiety disorder among occupational and physical therapists during treatment sessions and its association with somatic symptoms during the COVID-19 pandemic.

**Methods:**

A descriptive cross-sectional design was used in this study. Data were collected during the month of April 2021, and the study included occupational and physical therapists who practiced during COVID-19 from March 2020 to March 2021. The generalized anxiety disorder scale (GAD-7) and a modified patient health questionnaire (mPHQ-15) were used to examine self-reported anxiety and somatic symptoms among the study participants. The independent *t*-test was used to determine differences between groups based on GAD-7 and mPHQ-15 results. Spearman's correlation test and chi-squared test were used to find the relationships between different variables such as anxiety and somatic symptoms.

**Results:**

The study (*n* = 98 participants) included 56 occupational and 42 physical therapists. An 84% response rate was achieved. GAD-7 final score was μ = 9.21 ± 5.63 with 27% reporting no anxiety, 14% mild, 38% moderate, and 21% severe. Independent *t*-tests on GAD-7 scores showed significant differences between therapist specializations [*t*_(96)_ = −2.256; *p* = 0.026] and between therapists residing with or without their parents [*t*_(96)_ = −2.536; *p* = 0.013]. The mPHQ-15 final score was μ = 9.52 ± 5.54 with 13% reporting no symptoms (*n* = 13), 20% mild (*n* = 20), 38% moderate (*n* = 37), and 29% severe (*n* = 28). GAD-7 and mPHQ-15 scores were moderately positively correlated [*r*_(96)_ = 0.569; *p* <0.000]. The chi-squared test showed a significant association between GAD-7 levels of anxiety and mPHQ-15 levels of somatic symptoms [*x*^2^_(9, N = 98)_ = 70.62 *p* <0.000]. Therapists reported that the quality (76%) and effectiveness (20%) of their rehabilitation services were negatively impacted by the COVID-19 pandemic.

**Conclusion:**

The majority of study participants experienced moderate to severe anxiety and associated somatic symptoms. During COVID-19, ongoing psychological counseling of healthcare professionals such as occupational and physical therapists is required to maintain positive mental health. Implications for practice are presented.

## Introduction

Coronavirus disease 2019 (COVID-19) is a communicable respiratory tract disease that emerged in Wuhan, China in December 2019. The coronavirus spread rapidly and was declared a pandemic by the World Health Organization (1). COVID-19 continues to spread worldwide and by November 2021 over 250 million cases have been reported and over 5 million deaths (1). Healthcare organizations in every region have assembled all possible resources to deal with the pandemic. Overworked healthcare professionals caring for massive numbers of COVID-19 patients experienced extraordinary psychological stress related to high risk of infection amongst themselves as well as sickness and death among colleagues (2–4). The fact that the majority of healthcare professionals had no infectious disease expertise exacerbated the situation (4).

Kuwait is 17,820 square kilometers (6,880 square miles) in size with the population of 4,336,000 million (5). The first COVID-19 case in the country was recorded in February 2020 (6). The first wave of COVID-19 was observed between March and May 2020, and the second wave was observed between January and March 2021 (7). Between March 2020 and 2021, the number of COVID-19 cases was close to 200,000 (6). As the number of cases increased, local administrators assigned additional healthcare personals for treating and caring of COVID-19 patients. In addition, other areas of healthcare services, such as rehabilitation, continued to provide services to patients. Individual workloads increased due to strict COVID-19 precautionary measures such limitations on the number of workers per site and deactivating healthcare professionals with preexisting chronic diseases.

Anxiety is an emotional state with three interacting components: psychological, behavioral, and physical. Anxiety can be experienced at different levels of intensity, frequency, and duration of episodes (8). DSM-V generalized anxiety disorder (GAD) is defined as excessive anxiety and worry due to events or activities that occur most days over a period of at least 6 months. Excessive anxiety and worry may lead to impairment in social and occupational areas (9). Mallorquí-Bagué et al.s' review (8) highlights the link between anxiety and somatic symptoms. GAD is generally associated with developing somatic symptoms including headache, irritability, impaired concentration, sleep disturbance, fatigue, muscle and joint pain, and dizziness (8, 10–13). COVID-19 research indicates that moderate to severe levels of anxiety due to the pandemic is associated with somatic symptoms such gastrointestinal ailments, fatigue, and difficulty sleeping (10, 14, 15). Furthermore, when healthcare professionals interact with patients with or without COVID-19, they experience increased levels of anxiety and somatic symptoms (16–19).

Healthcare professionals have experienced varying levels of anxiety during the COVID-19 pandemic around the world (16–31). They were found to be positive for anxiety, depression, stress, insomnia, somatization symptoms, poor psychological wellbeing, and obsessive-compulsive symptoms (22, 29, 30). Moreover, young or female healthcare professionals were more inclined to psychological issues compared to older or male colleagues (16–18, 20, 22, 23, 30, 32). Close contact with COVID-19 patients was a major factor affecting levels of fear, anxiety, and depression (16, 19–21, 27, 30, 32, 33). In addition, Gündogmuş et al. (31) highlighted an increase in depression, anxiety, and stress during the second peak of the pandemic.

In the Arab world including Kuwait, COVID-19 research studies among healthcare professionals are limited, and more investigations need to be done (22). We found few studies that examined anxiety among healthcare professionals including physicians, nurses, allied health, and pharmacists in Kuwait (2), Oman (1), Saudi Arabia (6), United Arab Emirates (2), and Jordan (1) (20–26, 34–38). They concluded that healthcare professionals experienced significant level of anxiety ranging from 10 to 40% on the moderate to severe levels. Moreover, we found few studies that examined anxiety of the general population in Kuwait (1), Saudi Arabia (2), and Algeria (1) (21, 36, 39, 40). Similarly, they found high rate of anxiety among the general population. All the studies concluded that the pandemic had considerable impact on the mental health of healthcare professionals as well as the general populations. All the studies also recognized the importance of immediate psychological attention and intervention.

Additionally, we found a single study that directly investigated anxiety among physical therapists in South Korea (41), with no studies examining anxiety among occupational therapists explicitly. Insight into this area could help to gauge the level of anxiety among this population and its associated somatic symptoms, and possibly lead to recommendations for improving the mental health for those in need. Thus, the purpose of the study was to examine the level of anxiety among occupational (OTs) and physical therapists (PTs) who have interacted with patients throughout the COVID-19 pandemic.

## Materials and Methods

### Study Design

A descriptive cross-sectional design was used in this study. Variables relating to anxiety and physical symptoms were self-reported. Variables were measured on an ordinal scale.

### Participants

The target population was composed of OTs and PTs working in inpatient and outpatient settings of rehabilitation clinics in Kuwaiti governmental hospitals. Private sectors were not included because they would employ very limited number of OTs and PTs. Also, the logistics of approaching such sporadic centers were difficult. Inclusion criteria included therapists who worked during the COVID-19 pandemic from March 2020 to March 2021. The exclusion criteria included therapists that did not work during the specified period of the pandemic or had a history of anxiety disorder. Also, individuals with the history of anxiety disorders were excluded from the study because they would likely differ in their mental health characteristics than individuals without the history of anxiety disorders.

### Reporting Tools

The generalized anxiety disorder assessment (GAD-7) is a self-administered screening test to identify probable causes and severity of anxiety (42). GAD-7 is used with adults aged 18 years and older. It includes seven items on a 4-point Likert scale (0 = not at all, 1 = several days, 2 = more than half the days, 3 = nearly every day). Scoring ranges from 0 to 21 with scores of 5, 10, and 15 set as cut-off points for mild, moderate, and severe anxiety, respectively. Further evaluation is recommended when a score is 10 or greater.

The Patient Health Questionnaire (PHQ) is a self-administered version of the PRIME-MD diagnostic instrument for common mental disorders (43). PHQ-15 comprises 15 somatic symptoms from the PHQ. The 15 items are scored on 3-point Likert scale (0 = not bothered at all, 1 = bothered a little, and 2 = bothered a lot). The total PHQ-15 score ranges from 0 to 30 and scores of 5, 10, and 15 are set as the cut-off points for mild, moderate, and severe levels of somatization, respectively. However, due to cultural sensitivities, two items (question #4: menstrual cramps or other problems with your periods, and #11: pain or problems during sexual intercourse) were removed from the questionnaire. After adjustment, the modified PHQ-15 (mPHQ-15) was comprised of 13 somatic symptoms. The total mPHQ-15 score ranged from 0 to 26 and scores of 3, 18, and 13 were set as the cut-off points for mild, moderate, and severe levels of somatization, respectively. Clinical and occupational healthcare settings have demonstrated high reliability and validity for the PHQ-15 (44).

### Procedure

Ethical approval was obtained from the Institutional Review Boards of the local University Health Science Center and the Ministry of Health. The data collection was conducted during the month of April 2021. The departments heads sent the online survey link *via* WhatsApp and invited OTs and PTs to participate in the study. One week later, the departments heads were reminded to resend the link and to encourage participation in the study. Participants' names and phone numbers were only known to the departments heads, and no identifications of the respondents were included on the survey to insure anonymity. After reviewing the demographic data, participants who did not meet the inclusion criteria were excluded from the study. The entire survey was in English because all OTs and PTs in Kuwait must have had proficient English education background. The online survey included demographic information as well as GAD-7 and mPHQ-15 questionnaires. Therapists were instructed *via* the social media application that clicking on the link would indicate their consent to participate in the study. Following completion of the GAD-7 and mPHQ-15, the respondents were asked to answer two questions related to the quality (do you feel that the quality of therapy interventions was affected by the COVID-19 pandemic? (Yes/No) and effectiveness (how would you rate the effectiveness of rehabilitation interventions during the COVID-19 pandemic?) of the rehabilitation services during the COVID-19 pandemic. The entire survey took a maximum of 15 min to complete. This research protocol complied with the tenets of the Declaration of Helsinki.

### Data Analysis

The Statistical Package for the Social Sciences (SPSS) version 25 was used for analysis. Descriptive statistics were used to summarize demographics and questionnaire results. The independent *t*-test was used to determine differences between groups based on GAD-7 and mPHQ-15 results. Spearman's correlation test and chi-squared test were used to find the relationships between different variables. Cramer's *V*-test was used to determine level of association. A value of *p* <0.05 was set as the threshold for significance.

## Results

The survey was sent to 116 therapists and the response rate was 84%. The study included 98 participants (56 OTs; 42 PTs). Of these, 90 were female and 8 were male. The ages ranged from 22 to 52 years old with a mean of 31.86 ± 8.97 years. Years of experience ranged from 1 to 29 years with a mean of 8.08 ± 7.10 years. Participants' area of practice included pediatrics (*n* = 46), neurology (*n* = 36), and orthopedics (*n* = 16). Twenty-four percent of therapists contracted COVID-19, however, they were not certain on how they contracted the coronavirus (Table 1).

**Table 1:** Demographics of occupational and physical therapy participants.

	**OT**	**PT**	***N***
*N* (%)	56 (57%)	42 (43%)	98 (100%)
Infected	10 (18%)	13 (31%)	23 (24%)
**Gender**
Male	1 (12.5%)	7 (87.5%)	8 (8%)
Female	55 (61%)	35 (39%)	90 (92%)
**Area**
Neurology	12 (21%)	24 (57%)	36 (37%)
Pediatric	28 (50%)	18 (43%)	46 (47%)
Orthopedic	16 (29%)	00 (00%)	16 (16%)
**Age**
M ± SD	27.12 ± 5.11	38.17 ± 9.16	31.86 ± 8.97
**Experience**
M ± SD	4.23 ± 2.93	13.21 ± 7.78	8.08 ± 7.10

GAD-7 overall final score was μ = 9.21 ± 5.63 and its frequency distribution levels were 27% with no anxiety (*n* = 26), 14% mild (*n* = 14), 38% moderate (*n* = 37), and 21% severe (n=21; Table 2). For GAD-7, item #1 “feeling nervous, anxious or on edge” (47%), item #3 “worrying too much about different things” (56%), and item #6 “becoming easily annoyed or irritable” (42%) were the most reported anxiety symptoms with “more than half the days” and “nearly every day” responses (Table 3). The independent *t*-test showed significant differences between OTs and PTs for the GAD-7 scores [*t*_(96)_ = −2.256, *p* = 0.026] with PTs having a greater anxiety mean (μ = 10.67 ± 5.56) than OTs (μ = 8.13 ± 5.49). Also, the independent *t*-test on GAD-7 scores showed significant differences between therapists residing with their parents vs. therapists residing without their parents [*t*_(96)_ = −2.536; *p* = 0.013]. Therapists residing with their parents had greater anxiety (μ = 10.15 ± 5.92) than therapists residing without their parents (μ = 7.10 ± 4.20). The chi-squared test of independence showed a significant association between area of practice and GAD-7 levels [*x*^2^_(30, N = 98)_ = 50.88; *p* = 0.010]. Therapists working in the area of neurology had greater anxiety (μ = 10.64 ± 5.80) than therapists working in the areas of pediatrics (μ = 8.57 ± 5.23) or orthopedics (μ = 7.88 ± 6.00). Cramer's *V*-test showed a strong association between the variables (*V* = 0.509). However, based on GAD-7 scores there was no correlation with ages of therapists [*r*_(96)_ = 0.058; *p* = 0.572] and with years of experience [*r*_(96)_ = 0.076; *p* = 0.457].

**Table 2:** Levels of anxiety and somatic symptoms among the OT and PT respondents.

	**OT**	**PT**	**All**
	***n* = 56**	***n* = 42**	***n* = 98**
**Anxiety**
None	18 (32%)	8 (19%)	26 (27%)
Mild	9 (16%)	5 (12%)	14 (14%)
Moderate	19 (34%)	18 (43%)	37 (38%)
Severe	10 (18%)	11 (26%)	21 (21%)
**Somatic**
None	9 (16%)	4 (10%)	13 (13%)
Mild	12 (21%)	8 (19%)	20 (20%)
Moderate	20 (36%)	17 (40%)	37 (38%)
Severe	15 (27%)	31 (13%)	28 (29%)

**Table 3:** Occupational and physical therapists report on the high responses of GAD-7.

Item^*^	**Response**		**Cumulative %**
	**More than half the days (%)**	**Nearly every day (%)**	
1. Feeling anxious	27	15	42^b^
2. Not to stop worrying	28	12	40
3. Worrying too much	27	26	53^a^
4. Trouble relaxing	15	14	29
5. Restless that	10	8	18
6. Easily annoyed	21	20	41^c^
7. Felling afraid	24	15	39

* *Condensed version of the complete items*.

a, b, c *Most reported anxiety symptoms*.

The overall mPHQ-15 final score was μ = 9.52 ± 5.54, indicating an overall moderate level of somatization. The frequency distribution for mPHQ-15 was as follows: 13% of participants had no symptoms (*n* = 13), 20% mild (*n* = 20), 38% moderate (*n* = 37), and 29% had severe symptoms (*n* = 28; Table 2). For mPHQ-15, item #2 “back pain” (*n* = 39, 40%), item #11 “feeling tired or low energy” (*n* = 46, 47%), and item #12 “trouble sleeping” (*n* = 43, 44%) were the most reported somatic symptoms along with “bothered a lot” (Table 4). GAD-7 and mPHQ-15 overall scores were moderately positively correlated [*r*_(96)_ = 0.569; *p* < 0.000]. The chi-squared test of independence showed a significant association between GAD-7 levels of anxiety and mPHQ-15 levels of somatic symptoms [*x*^2^_(9, N = 98)_ = 70.62; *p* < 0.000]. Cramer's V test showed a strong association between the variables (*V* = 0.490).

**Table 4:** Occupational and physical therapists report on high responses of mPHQ-15.

Item^*^	**Bothered**	
	**A little %**	**A lot %**
1. Stomach pain	48	10
2. Back pain	45	40^c^
3. Joints, arm, leg pain	35	27
5. Headache	61	16
6. Chest pain	30	00
7. Dizziness	36	15
8. Fainting spells	18	03
9. Feeling heart pound	24	09
10. Shortness of breath	26	11
12. Constipation or diarrhea	38	07
13. Nausea or indigestion	47	11
14. Tired or low energy	33	47^a^
15. Trouble sleeping	32	44^b^

* *Condensed version of the complete items*.

*Items #4 and #11 were removed*.

a, b, c *Most reported bothered somatic symptoms*.

Our data showed that 76% of respondents thought that the quality of their rehabilitation services was negatively impacted by the COVID-19 pandemic. Also, when respondents were asked about the effectiveness of their rehabilitation services, 20% of them believed that their rehabilitation services were not effective. On the other hand, some respondents thought that the effectiveness of their rehabilitation services were the same (14%), somewhat effective (43%), or effective (23%).

## Discussion

This study showed that the majority of OTs and PTs who practiced during the COVID-19 pandemic in the State of Kuwait experienced moderate to severe GAD. However, our findings were not consistent with the South Korea's study, who reported milder level of anxiety PTs than our participants (41). Based on the DSM-V (9) definitions of moderate and severe anxiety, these occupational and physical therapists might experience social and occupational difficulties, and therapists with severe anxiety should receive professional medical intervention. Moreover, the respondents mainly experienced anxiety symptoms such as worrying too much, feeling anxious, annoyance and irritation, and feeling afraid due to the possibility of catching the coronavirus from patients attending rehabilitation services. Our findings paralleled the trends in other recent studies of the general population and healthcare professionals the Arab countries and around the world who reported having moderate to severe anxiety during the COVID-19 pandemic (10, 20–23, 29–31, 34, 45). Our results showed that neither the number of years of experience nor ages of the healthcare professionals had any influence on anxiety levels. Due to the unprecedented nature of COVID-19, therapists most likely were not prepared with adaptive strategies to cope with the new experience. However, this was not the case in other studies that were conducted in Kuwait, Oman, Saudi Arabia, United Arab Emirates, and Jordan, which found younger and female healthcare professionals to have experienced greater anxiety (20, 22, 23, 38).

Our findings showed that family living arrangements had an effect on the level of anxiety. Respondents residing with their parents reported greater anxiety symptoms on the GAD-7 questionnaire compared to respondents residing without their parents. This could be explained by the fact that therapists living with family were concerned about catching the coronavirus from patients and transmitting it to their parents, who were more likely to be older adults and, therefore, at greater risk for severe illness and/or death. Similar concerns among healthcare professionals have been reported in other studies (20, 23, 26, 46, 47).

In term of professions, PTs reported a significantly higher score on GAD-7 than OTs. A possible explanation is that PTs reported spending more time at work than OTs, which would increase their risk of contracting the coronavirus and consequently increase their level of anxiety. Pniak et al. (48) and Yang et al. (41) concluded that physical therapists experienced significant rise in occupational burnout and anxiety during the pandemic due to increase workload, and they suggested a possible emerging risk in mental health conditions. Also, the background knowledge of OTs in psychosocial sciences, mental health, and adaptation theory may also explain their reduced anxiety levels in comparison to PTs. In addition, therapists in the neurology field experienced more anxiety than therapists working in pediatric or orthopedic areas. A possible explanation may be that therapists working in the neurological setting might worry about COVID-19 infection due to dealing with patients with low immune systems, especially those in the intensive care or neuro-surgery units (49, 50).

According to results of the mPHQ-15, the majority of respondents experienced moderate to severe somatic symptoms. Also, a moderate positive correlation was found between GAD-7 and mPHQ-15 results indicating a simultaneous increase in somatic symptoms and anxiety during COVID-19 pandemic. These findings are reflective of pre- and post-COVID-19 research which shows that a relationship exists between anxiety and somatic symptoms (8, 14, 18, 51). Anxiety and somatic symptoms are common in the general population and can lead to anxiety disorders (8). Neuroimaging studies have provided greater insight into the understanding of anxiety and somatic symptoms, revealing how the amygdala, anterior cingulate cortex, and insula play key roles in the development and maintenance of anxiety symptoms (52, 53).

Healthcare professionals should be cautious when treating patients with somatic symptoms during the COVID-19 pandemic or other catastrophic events, since somatic symptoms may have psychological roots. Psychological targets for intervention can then be identified alongside basic biological mechanisms for anxiety (8). Therapists with somatic symptoms should take time off from work to lessen their symptoms, however, this would further stretch thin the number healthcare professionals available for patient care. Therefore, continuous psychological counseling for healthcare professionals might be necessary during these prolonged events of psychological distress.

### Implications and Future Studies

The majority of therapists indicated that the quality of rehabilitation services was negatively impacted by the COVID-19 pandemic. In addition, some therapists reported that their rehabilitation interventions were ineffective. Similarly, Hoel et al. (54) and Chimento-Díaz et al. (55) indicated that the majority of occupational therapists reported that their services were less effective and with lower quality during the pandemic. Due to precautionary procedures and measures, a number of rehabilitation services and activities were temporary discontinued during the pandemic, and home exercise programs were increased to reduce contact with patients. We recommend that healthcare education programs and future conferences and seminars should focus on the use of technology for communication and medical intervention. For example, telerehabilitation can be expanded which includes the use of videoconferencing, phones, email, and apps for individual or group remote therapy sessions. Kreider et al. (56) highlighted that telerehabilitation improved the patients' health and rehabilitation experience in addition to providing convenience, privacy, and comfort. They pointed out that telerehabilitation developed greater active participation and empowerment of the patients to carry out the rehabilitation programs. Also, virtual reality therapy, which is a computer program that creates an artificial environment to give the patient a simulated experience, can allow patients to continue their treatment at home and at the same time it has the benefit of significantly decreasing direct therapist-patient contact. Ilyas et al. (57), who examined patients' visits to the rehabilitation services during the pandemic, recommended improving rehabilitation programs through the use of patient-centered approach. Thus, technology can help in in maintaining rehabilitation services, reducing contact between therapists and patients, slowing the spread of the virus, and protecting vulnerable populations.

Based on our findings, we recommend that psychological counseling should be required for frontline healthcare providers during pandemics and catastrophic events. Also, it is imperative that healthcare workers engage in continuous training and preparation for future events. They must learn coping strategies that support the management of anxiety and psychological distress that is inevitably encountered during such experiences. Organizations and institutions should be required to offer positive support, to promote resilience, and to educate employees in adaptation/coping strategies (20, 58, 59). Doing so would reduce encountered stress and psychological distress and lead to better healthcare delivery (47).

Future studies are needed to examine the effectiveness of courses, training strategies, and technologies that target preventative measures for healthcare professionals in order to ensure a higher level of readiness for tackling pandemics. As different variants of COVID-19 arise, level of stress among healthcare professionals increases due to uncertainty of consequences, rapidity of spread, contiguousness, and strict precautionary measures (58). Therefore, public health officials must be prepared with ready guidelines, updated information, and recommendations to deal with emerging new variants. Such measures can help to support healthcare professionals to minimize their anxiety and fear, and consequently improve their confidence in dealing with pandemic related issues (58, 60). Administrators must be retrained and given greater responsibilities to monitor the status of healthcare workers during pandemics in order to achieve healthier working environments and support desired therapeutic outcomes for different patient populations. Moreover, more research is needed to optimize strategies for reducing anxiety levels and associated somatic symptoms during such events. Finally, healthcare protocols must detail the guidelines to protect the good health, prevent spread of illness, and maintain quality of care and services.

## Limitations

This study had some limitations. The sample of OTs and PTs included a great imbalance between males and females. Therefore, we were unable to study gender differences on anxiety. The research study had a small number of participants. Although, 98 participants provided enough data to reveal trends, a larger number would show more robust results. In addition, our findings might have limited generalizability to all healthcare professions. However, our findings were similar to the findings of other research studies with different populations in various countries. In addition, our online survey was subject to response bias as respondents with a notion of psychological distress might have been more likely to complete the survey.

## Conclusion

The COVID-19 pandemic caused a significant impact on mental health of healthcare professionals such as occupational and physical therapists. This study provided important results for the association between anxiety and somatic symptoms. The majority of study participants experienced moderate to severe anxiety and associated somatic symptoms. During crisis events such as the COVID-19 pandemic, ongoing psychological counseling of healthcare professionals is required to maintain positive mental health and to minimize associated somatic symptoms. Interventions are needed to support healthcare professionals by focusing on both the psychological manifestations and physical symptoms.

## Data Availability Statement

The original contributions presented in the study are included in the article/supplementary material, further inquiries can be directed to the corresponding author.

## Ethics Statement

The studies involving human participants were reviewed and approved by Health Sciences Center, Kuwait University and Kuwait Ministry of Health. The patients/participants provided their written informed consent to participate in this study.

## Author Contributions

MA, NA, MN, FM, and HA: conceptualization, methodology, and resources. MA and HA: validation. MA, NA, MN, and FM: formal analysis. MA: writing—original draft preparation. NA, MN, FM, and HA: reviewing and editing. All authors have read and agreed to the published final version of the manuscript.

## Conflict of Interest

The authors declare that the research was conducted in the absence of any commercial or financial relationships that could be construed as a potential conflict of interest.

## Publisher's Note

All claims expressed in this article are solely those of the authors and do not necessarily represent those of their affiliated organizations, or those of the publisher, the editors and the reviewers. Any product that may be evaluated in this article, or claim that may be made by its manufacturer, is not guaranteed or endorsed by the publisher.
